# Prolonged Activation of Brain CB2 Signaling Modulates Hypothalamic Microgliosis and Astrogliosis in High Fat Diet-Fed Mice

**DOI:** 10.3390/ijms23105527

**Published:** 2022-05-16

**Authors:** Rodrigo Rorato, Nathalia L. Ferreira, Franciane P. Oliveira, Higor J. Fideles, Tays A. Camilo, Jose Antunes-Rodrigues, Andre S. Mecawi, Lucila L. K. Elias

**Affiliations:** 1 Laboratory of Stress Neuroendocrinology, Department of Biophysics, Paulista Medical School, Federal University of Sao Paulo, Sao Paulo 04023-062, Brazil; ferreira.nathalia@unifesp.br (N.L.F.); fpoliveira@unifesp.br (F.P.O.); higorjose66@hotmail.com (H.J.F.); 2 Department of Physiology, Ribeirao Preto Medical School, University of Sao Paulo, Ribeirao Preto 14049-900, Brazil; antunes@fmrp.usp.br; 3 Laboratory of Molecular Neuroendocrinology, Department of Biophysics, Paulista Medical School, Federal University of Sao Paulo, Sao Paulo 04023-062, Brazil; camilo.tays@unifesp.br (T.A.C.); mecawi@unifesp.br (A.S.M.)

**Keywords:** obesity, CB2 signaling, hypothalamic gliosis, glycaemia

## Abstract

Low-grade inflammation of the hypothalamus is associated with the disturbance of energy balance. The endocannabinoid system has been implicated in the development and maintenance of obesity as well as in the control of immune responses. The type 2 cannabinoid receptor (CB2) signaling has been associated with anti-inflammatory effects. Therefore, in high fat diet (HFD)-induced obese mice, we modulated CB2 signaling and investigated its effects on energy homeostasis and hypothalamic microgliosis/astrogliosis. We observed no effect on caloric intake and body weight gain in control diet-fed animals that received prolonged icv infusion of the CB2 receptor agonist HU308. Interestingly, we observed a decrease in glucose tolerance in HFD-fed animals treated with HU308. Prolonged icv infusion of HU308 increases astrogliosis in the ventromedial nucleus (VMH) of obese animals and reduced HFD-induced microgliosis in the hypothalamic arcuate (ARC) but not in the paraventricular (PVN) or VMH nuclei. These data indicate that central CB2 signaling modulates glucose homeostasis and glial reactivity in obesogenic conditions, irrespective of changes in body weight.

## 1. Introduction

Obesity is a chronic disease that has been reported to reduce the life expectancy and quality of life of affected individuals [1]. This disease is associated with the development of several comorbidities, such as type 2 diabetes mellitus, fatty liver disease, hypertension, myocardial infarction, stroke, Alzheimer’s disease and depression [2]. Obesity is primarily caused by an energy imbalance between calories consumed and energy expenditure [3]. Over the last 2–3 decades, studies have concentrated on identifying the areas of the central nervous system and mechanisms/circuits involved in the regulation of food intake and energy balance.

The hypothalamus is a key area of the brain for controlling energy homeostasis [4]. In particular, several studies have investigated the role of the hypothalamic ventromedial (VMH), arcuate (ARC) and paraventricular (PVN) nuclei in the control of food intake, energy expenditure and glucose homeostasis [4,5]. Initial studies in this area that used targeted electrolytic lesions indicated that the VMH is an important anorexigenic site [6]. However, recently, the role of the VMH in the control of energy and glucose homeostasis has been investigated using newer and more specific techniques of neuronal modulation and heterogeneous neuronal cell types and their responsivity to peripheral signals that are involved in the control of energy homeostasis have been reported [7]. Regarding the ARC, although transcriptome studies have revealed the existence of different cell clusters [8], the ARC nucleus is still classically defined as expressing two important groups of neurons. One group expresses the anorexigenic precursor proopiomelanocortin (POMC) and the other group coexpresses the orexigenic agouti-related protein (AgRP) and neuropeptide Y (NPY) [4,9]. Changes in the activity of ARC neurons have a profound impact on glucose and energy homeostasis [4,5]. The ARC neurons directly project to the PVN and exert their metabolic and behavioral effects, in part, through changes in the activity of PVN neurons expressing melanocortin receptor 3/4 [9,10]. The PVN is essential to the control of the endocrine axis, which is involved in the control of growth and development, metabolism and response to stress [11,12,13].

It is well known that the consumption of a high-fat diet (HFD) impairs the activity of hypothalamic neurons, especially ARC neurons. This diet causes insensitivity to peripheral signals that are related to changes in the peripheral energy stores that in turn can lead to different metabolic diseases [14]. HFD-induced obesity is associated with the development of low-grade inflammation in the hypothalamus [15]. Interestingly, a previous study reported that low-grade inflammation of the hypothalamus occurs before changes in peripheral energy stores [16]. The increased expression of cytokines in the hypothalamus is associated with microgliosis and astrogliosis [17]. In fact, a study in HFD-fed animals suggested that astrocytes and microglia initially inhibited hypothalamic inflammatory signaling. However, prolonged exposure activated glial cells and eventually induced a neurotoxic phenotype [16].

Considering hypothalamic low-grade inflammation is an important event that is associated with the development of obesity, several groups have investigated different molecules with pro/anti-inflammatory properties as potential targets to treat obesity. In this sense, in addition to its classical function as an orexigenic factor that is mediated by cannabinoid type 1 receptor (CB_1_) signaling [18], endocannabinoids have been associated with important effects on inflammation through the modulation of cannabinoid type 2 receptor (CB_2_) signaling. The high expression of the CB_2_ receptor in brain immune cells reinforces the involvement of the endocannabinoid system in the control of the inflammatory response [19,20]. Indeed, tetrahydrocannabinol or anandamide reduces lipopolysaccharide-induced interleukin 6 (IL-6) and tumor necrosis alpha (TNF-α) secretion in macrophage/microglia cell culture [21,22]. In addition, the increase in proinflammatory cytokines in the prefrontal cortex of mice exposed to immobilization stress can be prevented by specific CB_2_ agonism [23].

Modulation of CB_2_ signaling can also affect energy and glucose homeostasis. It was reported by Verty and colleagues (2015) [24] reduced food intake in lean and obese animals after the administration of JWH-015, which is a CB_2_ agonist. Regarding the modulating effects of CB_2_ signaling on glycemia, acute activation of the CB_2_ receptor has been shown to improve glucose tolerance in lean animals [25]. However, an increase in HFD-induced insulin resistance was also observed after the administration of a CB_2_ agonist [26]. Despite the existence of some contradictory results, these studies demonstrate the role of CB_2_ signaling in the modulation of food intake and glucose homeostasis. However, it is still not clear whether the central nervous system is involved in these responses.

Therefore, in the present study, we aimed to investigate the effects of the prolonged intracerebroventricular (icv) modulation of CB_2_ receptor signaling with HU308 on HFD-induced hypothalamic micro/astrogliosis and its consequence on energy and glucose homeostasis.

## 2. Materials and Methods

### 2.1. Animals

All procedures were approved by the Ethical Committee for Animal Use of the Ribeirao Preto Medical School (140/2012). Male C57BL mice (7–8 weeks of age) were obtained from the Central Animal Facility of the University of Sao Paulo-Ribeirao Preto Campus. The animals were housed in groups under controlled light (12:12 h light-dark cycle; lights off at 6:00 PM) and temperature conditions (23 ± 2 °C), with free access to water and food, unless otherwise stated.

### 2.2. Experimental Procedures

Mice were assigned to 4 groups (n = 6–8/group): (1) control diet + vehicle, (2) control diet + HU308, (3) HFD + vehicle and (4) HFD + HU308. Animals were fed a control diet or HFD for approximately 56 days. Two weeks before the end of the experiment, mice were subjected to subcutaneous minipump (Model 1002, ALZET, San Diego, CA, USA) + brain infusion Kit (Brain Infusion Kit 3, ALZET) implantation for continuous icv infusion of a vehicle or HU308 (5 ug/day—rate of 208.3 ng/h) [27]. The brain infusion cannula was implanted into the lateral ventricle (coordinates—AP: 0.1 mm, lateral: 1.0 mm and DV: 2.6 mm from the bregma) of the animals. HU308, a potent and selective CB_2_ receptor agonist (Hanus et al. 1999), was acquired from Tocris (Bristol, UK). It was initially resuspended in 1:1 ethanol and cremophor EL and later diluted to 1:50 in 0.9% saline.

During the first and second weeks of continuous icv infusion of the drugs, we measured body weight changes and food intake. Ten days after the cannula was implanted, a glucose tolerance test (GTT) was performed. On the day after the last body weight and food intake measurements, the animals were anaesthetized and perfused (10:00 AM to 12:00 AM) for brain collection for immunochemistry studies.

### 2.3. Assessment of Food/Caloric Intake, Body Weight Gain, the Food Efficiency Ratio and the Glucose Tolerance Test

Before implanting the osmotic minipumps, the animals were kept in individual cages and handled weekly for adaptation to the experimental procedures for food intake and body weight gain measurements. The animals were fed a control diet (3.85 kcal/g; 10% kcal fat, 20% kcal protein and 70% kcal carbohydrate) or HFD (Research Diets—5.24 kcal/g; 60% kcal fat, 20% kcal protein and 20% kcal carbohydrate; D12492; Research Diets Inc., New Brunswick, NJ, USA) throughout the experimental period, which was approximately 56 days. Two weeks before the end of the experiment, the animals underwent stereotaxic surgery for osmotic minipump implantation. The locomotor activity, food intake, urine and feces output, hydration, pain (measured during cage changes) and the surgery area were monitored during the 2 weeks post-surgical to assess the welfare of the animals.

After surgery, we assessed the body weight changes and the average weekly food intake. Body weight and food intake measurements were performed between 08:00 AM and 12:00 AM. The GTT was performed 10 days after the initiation of brain infusion from 12:00 AM to 2:00 PM. Food was removed at 08:00 AM and the animals were placed in a clean cage. Then, they received an intraperitoneal (ip) administration of glucose (2 mg/g) and their glycemia was measured at 0, 15, 30 60, 90 and 120 min from a drop of blood obtained from a small incision in their tail.

The caloric intake was determined based on the caloric value (kcal/g) of each diet and the food efficiency ratio during the first and second weeks of treatment was calculated by applying the following equation: [body weight gain (mg)/food intake (kcal) × 100].

### 2.4. Transcardiac Perfusion, Tissue Collection and Immunostaining

Two weeks after the surgery, the animals were deeply anaesthetized with a mixture of ketamine and xylazine (ketamine 45 mg/kg body weight and xylazine 5 mg/kg body weight) and perfused with a transcardiac infusion of 0.9% phosphate-buffered saline (PBS 1X) followed by 10% neutral buffered formalin solution (Sigma–Aldrich, Taufkirchen, Germany). At the end of the perfusion, the brain was removed. It was postfixed in the same fixative for 4 h and equilibrated in 30% sucrose. Then, 30 µm coronal sections were obtained in a cryostat.

The brain sections were used to perform single immunostaining for ionized calcium binding adaptor molecule 1 (Iba1-microglia), glial fibrillary acidic protein (GFAP-astrocytes), phospho-signal transducer and activator of transcription 3 (pSTAT3-leptin signaling) and phospho-extracellular signal-regulated kinase (pERK). The brain sections were rinsed with 1X PBS and incubated in 5% normal donkey serum (Jackson ImmunoResearch Laboratories, Inc., West Grove, PA, USA) and 0.3% Triton for 1 h. The slices were then incubated overnight at room temperature with rabbit anti-Iba1 (1:1000, WAKO, Richmond, VA, USA), rabbit anti-GFAP (1:1000, ZYMED/Thermo Fisher Scientific, Waltham, MA, USA) or rabbit anti-pERK (1:1000, Cell Signaling Technologies, Danvers, MA, USA), or they were incubated for 48 h at 4 °C with rabbit anti-pSTAT3 (1:500, Cell Signaling Technologies). After rinsing, the sections were incubated with the secondary biotinylated antibody donkey anti-rabbit (1:400, Vector Laboratories, Burlingame, CA, USA) and processed using the Vectastain Elite avidin-biotin (ABC) immunoperoxidase method (Vector Laboratories). We used the DAB-Peroxidase substrate kit (Vector Laboratories) to reveal the immunostaining. Finally, the sections were mounted on slides, air-dried overnight, cleared in xylene and placed under a coverslip with Entellan.

Immunopositive cells were identified in hypothalamic regions according to the following coordinates from the atlas of Franklin and Paxinos (2008) [28]: PVN: −0.92 mm to −2.12 mm from bregma; ARC and VMH: −2.3 mm to −3.5 mm from bregma. Photomicrographs were captured with a Leica microscope equipped with a DC 200 digital camera, attached to a contrast enhancement device. Two or three images from each animal were quantified using the software FIJI (version 1.5; WS Rasband, National Institute of Health, Bethesda, MD, USA). A region of interest (ROI) (images of 20× magnification) in the PVN, ARC and VMH was delimited according to its cytoarchitecture, allowing for the quantification of a constant area in all samples. For the analysis of Iba1+ microglia, the number of somata was quantified. The average pixel intensity (inch^2^) of the ROI was used to quantify GFAP immunostaining. The number of immunoreactive-positive cells for pSTAT3 or pERK was obtained by counting the black (nuclear) staining. All immunostaining analyses were performed in a blinded fashion with no access to the group/number of the animals or other data previously obtained.

### 2.5. Statistical Analysis

The data are expressed as the mean ± SEM. Samples were tested for a regular distribution using the Shapiro–Wilk normality test (Prism 9.0-GraphPad software, San Diego, CA, USA) and for the presence of outliers (Grubb’s test). The AUC of GTT study were analyzed through an unpaired *t* test. For the other results, we used two-way ANOVA followed by Šídák (only for GTT) or Tukey’s post-hoc tests to detect statistical significance. Differences were accepted as significant at *p* < 0.05.

## 3. Results

### 3.1. Glucose Tolerance Is Reduced in HFD-Fed Animals during the Prolonged Brain CB_2_ Receptor Activation

**Figure 1:**
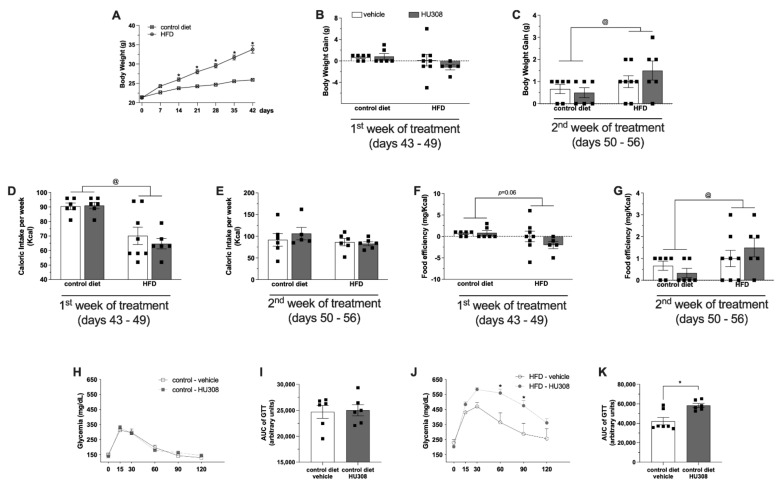
Effect of prolonged icv infusion of vehicle or HU308 for 2 weeks on the body weight, caloric intake, food efficiency ratio and GTT in animals fed a control diet or high-fat diet (HFD). Body weight curve in control diet-fed or HFD-fed animals before surgical procedure (**A**). The body weight gain (g) (**B**,**C**), caloric intake (Kcal) (**D**,**E**) and food efficiency (mg/Kcal) (**F**,**G**) after 1 or 2 weeks of icv vehicle or HU308 infusion in control diet-fed or HFD-fed animals. Plasma glucose levels (mg/dL) after glucose overload in control diet-fed (**H**) or HFD-fed (**J**) animals during prolonged infusion of vehicle or AM630. AUG of GTT in control diet-fed (**I**) or HFD-fed (**K**) animals treated with vehicle or AM630. One sample from HFD + vehicle group in the GTT study were not used because of problems with glucose overload injection. The data are expressed as the mean ± SEM; n = 5–8 per group. Data were analyzed by unpaired *t* test (**I**,**K**) or two-way ANOVA followed by Tukey’s (**B**–**G**) or Šídák’s (**H**,**J**) post-hoc test. @ *p* < 0.02 (effect of diet), * *p* = 0.05 (effect of HU308).

We examined the body weight, caloric intake, food efficiency ratio and glucose tolerance in control diet-fed or HFD-fed animals treated with icv infusion of a vehicle or HU308 for two weeks. The body weight of mice fed a HFD was increased compared to the body weight of mice fed the control diet (Figure 1A, * *p* < 0.05). We observed no difference in body weight gain between control diet-fed and HFD-fed mice after the first week of icv treatment (Figure 1B). However, two-way ANOVA showed significant effects of the diet (*p* = 0.03) on body weight gain after the second week of vehicle or drug infusion (Figure 1C). Two-way ANOVA also revealed a significant effect of the diet on caloric intake (*p* < 0.001) only in the first week of treatment. The calorie consumption was increased in animals fed a regular diet compared with animals fed a HFD (Figure 1D,E). A main effect of diet that was indicated by the two-way ANOVA was the effect on the food efficiency ratio (*p* = 0.03) in the second week of treatment. The food efficiency ratio in animals fed a HFD was increased compared with the animals fed a control diet (Figure 1G).

Regarding the glucose tolerance test, we observed no difference in the GTT (Figure 1H) or in the AUC of the GTT (Figure 1I) between control diet-fed animals treated with vehicle or HU308. Intriguingly, we observed that HU308 administration reduced the glucose tolerance of HFD-fed animals (60 min: *p* = 0.001, 90 min: *p* = 0.002) (Figure 1J,K).

### 3.2. The Prolonged Brain Activation of CB_2_ Receptor Reduced Microgliosis in the ARC of HFD-Fed Mice

We also investigated the effects of HU308 infusion on microgliosis in the hypothalamus of animals fed a control diet or HFD.

**Figure 2:**
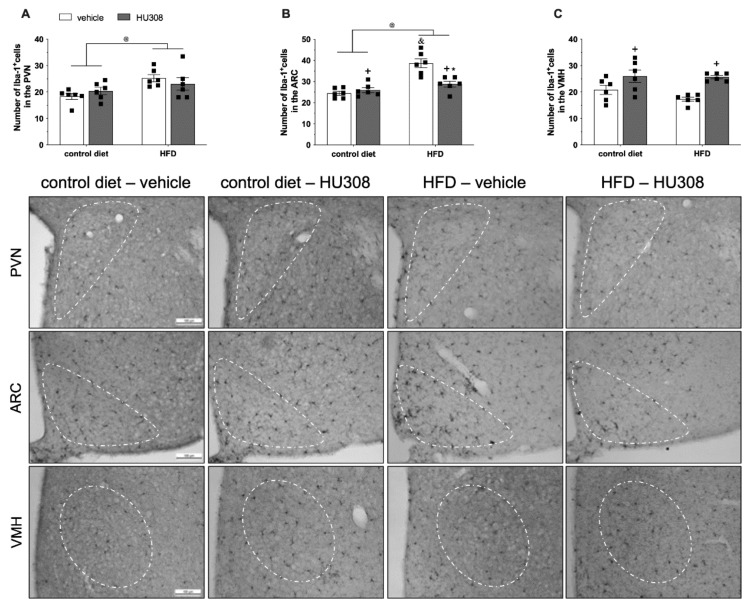
Effect of prolonged icv infusion of vehicle or HU308 for 2 weeks on the number of Iba-1+ cells in the paraventricular (PVN), arcuate (ARC) and ventromedial (VMH) nuclei in animals fed a control diet or high-fat diet (HFD). Number of Iba-1+ cells in the PVN (**A**), ARC (**B**) and VMH (**C**) nuclei in animals fed a control diet or HFD that received icv injection of vehicle or HU308. The data are expressed as the mean ± SEM; n = 6 per group. Data were analyzed by two-way ANOVA followed by Tukey’s post-hoc test. @ *p* < 0.05 (effect of diet), + *p* = 0.01 (effect of treatment), * *p* < 0.001 vs HFD+vehicle and & *p* < 0.001 vs control diet+vehicle. Bottom panel: Representative photomicrographs of Iba1 immunostaining in the PVN (superior panel), ARC (central panel) and VMH (inferior panel) of control diet-fed or HFD-fed animals treated with vehicle or HU308.

Regarding microglial immunostaining, we observed an effect of diet on the number of Iba1+ cells in the PVN (Figure 2A). There was a higher number of Iba1+ cells (*p* = 0.007) in HFD-fed animals compared to the control diet-fed animals. In the ARC, two-way ANOVA revealed an effect of diet (*p* < 0.0001), treatment (*p* = 0.01) and, an interaction (*p* = 0.001) between the groups on the number of Iba1+ cells. Interestingly, the post-test indicated that the continuous infusion of HU308 in HFD-fed animals reduced (*p* < 0.001) the number of Iba1+ positive cells in the ARC compared to HFD-fed animals treated with the vehicle (Figure 2B). We also observed an effect of treatment (*p* < 0.001) according two-way, with increased number of Iba1+ cells in the VMH (*p* = 0.005) of the HFD-HU308 group compared to the HFD-vehicle group (Figure 2C).

### 3.3. The Prolonged CB_2_ Receptor Activation Increased Astrogliosis in the VMH of HFD-Fed Mice

Considering the effects of CB_2_ signaling on inflammatory responses and the role of astrocytes in the control of extracellular homeostasis, we evaluated the effects of HU308 infusion on astrogliosis in the hypothalamus of animals fed a control diet or HFD.

**Figure 3:**
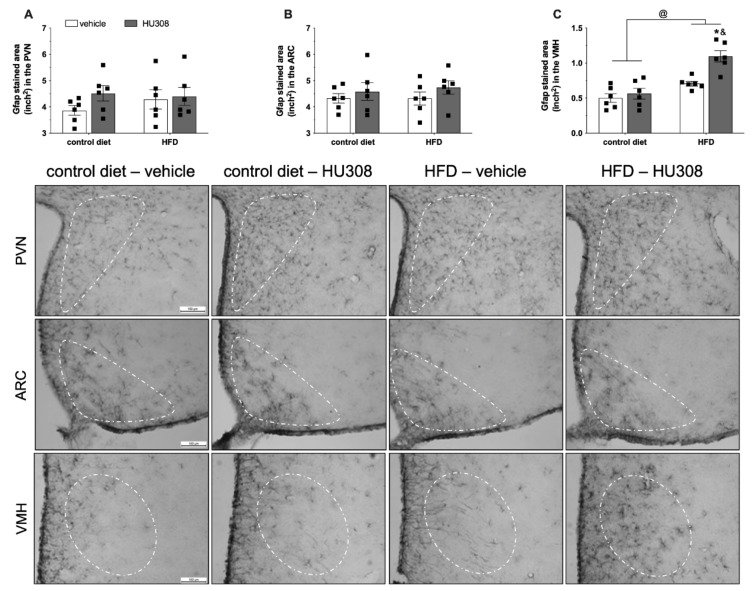
Effect of prolonged icv infusion of vehicle or HU308 for 2 weeks on the GFAP-stained area (inch2) in the paraventricular (PVN), arcuate (ARC) and ventromedial (VMH) nuclei in animals fed a control diet or high-fat diet (HFD). The GFAP-stained area (inch2) in the PVN (**A**), ARC (**B**) and VMH (**C**) nuclei in animals fed a control diet or HFD that received icv injection of vehicle or HU308. The data are expressed as the mean ± SEM; n = 6 per group. Data were analyzed by two-way ANOVA followed by Tukey’s post-hoc test. @ *p* < 0.001 (effect of diet), & *p* < 0.001 vs control diet+HU308 and, * *p* < 0.001 vs HFD+vehicle. Bottom panel: Representative photomicrographs of GFAP immunostaining in the PVN (superior panel), ARC (central panel) and VMH (inferior panel) of control diet-fed or HFD-fed animals treated with vehicle or HU308.

We observed no difference in GFAP expression in the PVN (Figure 3A) and ARC (Figure 3B) between the groups. In addition, we observed an effect of diet (*p* < 0.001), treatment (*p* = 0.002) and, an interaction (*p* = 0.02) between the groups on GFAP expression in the VMH. The post-test revealed increased GFAP staining in the VMH of HFD-fed animals treated with HU308 when compared to control diet-fed animals treated with HU308 (*p* < 0.001) and HFD-fed animals treated with the vehicle (*p* = 0.002) (Figure 3C).

### 3.4. The Absence of the Effects of Prolonged Brain CB_2_ Receptor Activation on STAT3 and ERK Signaling

The energy homeostasis control and inflammatory signaling processes recruit different intracellular signaling pathways, including STAT3 and ERK phosphorylation. Animals with diet-induced obesity have reduced STAT3 phosphorylation in the ARC in response to exogenous leptin administration or inflammatory stimuli and increased basal pSTAT3 in the ARC and VMH [14,29]. Increased ERK phosphorylation is involved in the thermogenic leptin effects and is also recruited after CB_2_ receptor activation [30,31]. Therefore, considering the role of both proteins in energy and glucose homeostasis control and their recruitment during the inflammatory process, we also investigated whether the modulation of CB_2_ signaling could modulate STAT3 and ERK phosphorylation in hypothalamic nuclei in lean and obese mice.

**Figure 4:**
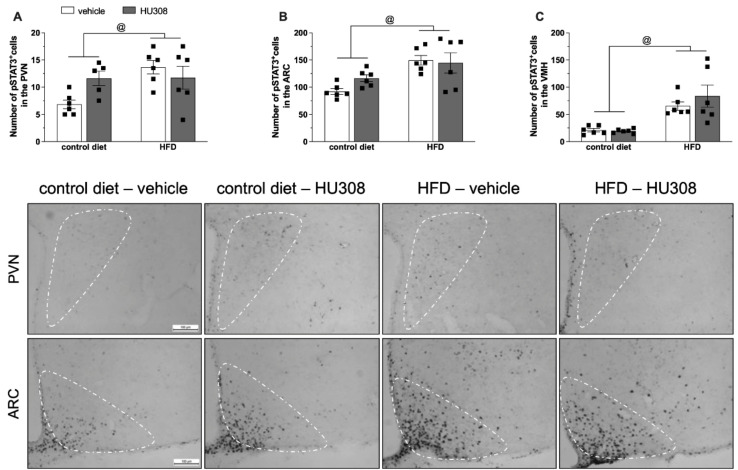

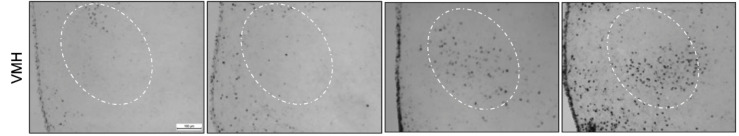
Effect of prolonged icv infusion of vehicle or HU308 for 2 weeks on the number of pSTAT3+ cells in the paraventricular (PVN), arcuate (ARC) and ventromedial (VMH) nuclei in animals fed a control diet or high-fat diet (HFD). Number of pSTAT3+ cells in the PVN (**A**), ARC (**B**) and VMH (**C**) nuclei in animals fed a control diet or HFD that received icv injection of vehicle (white bars) or HU308 (dark grey bars). The data are expressed as the mean ± SEM, n = 5–6 per group. One sample from control diet + HU308 group were missed during the pSTAT3 immunostaining protocol. Data were analyzed by two-way ANOVA followed by Tukey’s post-hoc test. @ *p <* 0.05 (effect of diet) Bottom panel: Representative photomicrographs of pSTAT3 immunostaining in the PVN (superior panel), ARC (central panel) and VMH (inferior panel) of control diet-fed or HFD-fed animals treated with vehicle or HU308.

The two-way ANOVA revealed a significant effect of the diet on pSTAT3 expression in the PVN (*p* = 0.02), ARC (*p* = 0.001) and, VMH (*p* < 0.001) (Figure 4A–C, respectively), with increased pSTAT3 in HFD-fed animals compared to control diet group. The continuous infusion of HU308 induced no change in STAT3 phosphorylation in the PVN, ARC or VMH.

**Figure 5:**
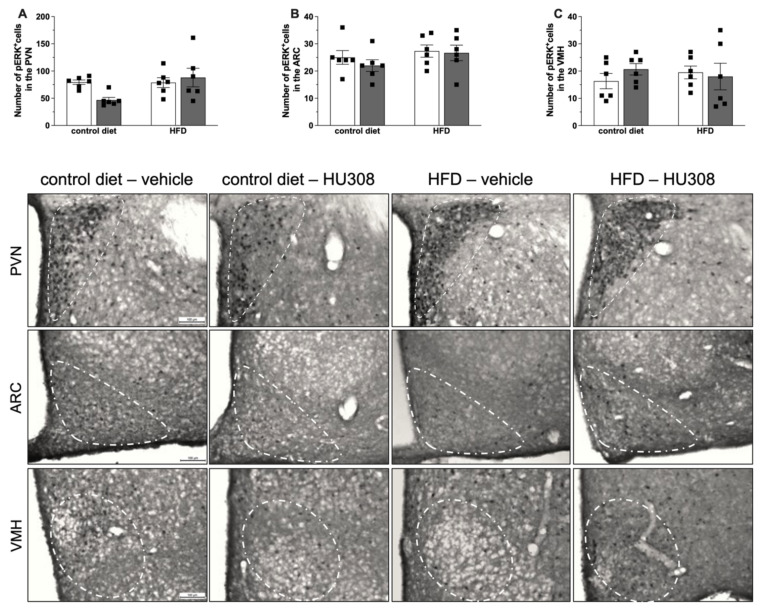
Effect of prolonged icv infusion of vehicle or HU308 for 2 weeks on the number of pERK+ cells in the paraventricular (PVN), arcuate (ARC) and ventromedial (VMH) nuclei in animals fed a control diet or high-fat diet (HFD). Number of pERK+ cells in the PVN (**A**), ARC (**B**) and VMH (**C**) nuclei in animals fed a control diet or HFD that received icv injection of vehicle or HU308. The data are expressed as the mean ± SEM, n = 6 per group. Data were analyzed by two-way ANOVA followed by Tukey’s post-hoc test. Bottom panel: Representative photomicrographs of pERK immunostaining in the PVN (superior panel), ARC (central panel) and VMH (inferior panel) of control diet-fed or HFD-fed animals treated with vehicle or HU308.

Additionally, we observed no difference in the pERK immunostaining in the PVN, ARC or VMH among all the groups (Figure 5A–C).

## 4. Discussion

Previous studies that have investigated the consequences of CB_2_ receptor signaling modulation have preferentially used the intraperitoneal route for drug administration or a global CB_2_ receptor knockout mice. However, these approaches preclude the analysis of whether the effects of CB_2_ receptor signaling modulation on energy and glucose homeostasis are mediated by central or peripheral actions. Therefore, to overcome this limitation, we used minipumps to deliver the CB_2_ receptor agonist HU308 directly to the brain. The main finding of our study was the reduction in microgliosis in the ARC and the increase in astrogliosis of HFD-fed animals that received a continuous infusion of HU308. However, we observed no effect of prolonged icv infusion of HU308 on body weight gain or caloric intake in either the control or HFD groups. Interestingly, the continuous infusion of this drug reduced glucose tolerance in HFD-fed mice.

It was also reported that deletion of the CB_2_ receptor gene reduced HFD-induced weight gain and it reduced the adipose and liver inflammation associated with obesity [26]. However, overexpression of CB_2_ receptors in the brain was also associated with decreased food intake and a lean phenotype [32] and the deletion of the CB_2_ receptor gene increased body weight [33,34]. CB_2_ receptor deletion in age-related obesity is associated with the proinflammatory polarization of immune cells in adipocytes and the liver and high mortality [34]. Despite these contradictory results on the association between energy homeostasis and CB_2_ receptor deletion or overexpression, these studies strongly suggest that CB_2_ signaling is involved in the regulation of food consumption and body weight and that changes in inflammatory response underlie these effects.

Pharmacological studies also support the involvement of CB_2_ signaling in energy homeostasis control. We observed no effect of the central modulation of CB_2_ signaling with HU308 treatment on body weight and caloric intake. However, it was reported that the peripheral administration of AM630, an inverse-agonist of CB_2_ receptor, can inhibit nocturnal food intake, but it also reduces hyperphagia after fasting [35]. In another study using peripheral AM630 administration, no change in body weight was observed in obese *ob/ob* animals, but there was reduced inflammation in the adipose tissue [26]. Furthermore, the use of the CB_2_ agonist HU308 for three months as drug-enriched pellets induced no effect on HFD-induced obesity, except for reduced macrophage polarization in the liver and adipose tissue [34]. The peripheral administration of a CB_2_ agonist (JWH-133) also induced no effect on body weight but potentiated white adipose tissue inflammation [26]. Importantly, low-grade inflammation in the periphery and brain is associated with obesity [15,36]. The existence of several contradictory results about the role of CB_2_ signaling on energy homeostasis and obesity-associated inflammation can be associated with the use of CB_2_ receptor-deleted animals, as well as to the existence of several differences between the experimental protocols performed by the different researchers. However, we must consider that several studies in the literature have reported an important anti-inflammatory function of CB_2_ signaling activation via agonists, inverse agonists and the use of transgenic animals with hyperexpression see reviews [37,38]. Therefore, more studies are needed to better understand the endocannabinoid function of CB_2_ signaling on energy homeostasis during inflammation caused by HFD consumption.

The modulation of CB_2_ receptor signaling also affects glucose metabolism. Interestingly, in our study, we observed reduced glucose tolerance in HFD-fed animals that received prolonged icv infusion of HU308. Similar to our results, a previous study reported that ip administration of JWH-133 for 15 days potentiated HFD-induced insulin resistance [26]. Furthermore, they demonstrated that the deletion of the CB_2_ receptor improved insulin sensitivity in HFD-fed animals [26]. Additionally, it was shown that the glycemia of mice with CB_2_ receptor gene deletion that were fed a regular diet was normal and that the hypoglycemia during ITT was more pronounced in these animals [33]. However, under a HFD regimen, the insulin sensitivity and plasma glucose levels were similar between CB_2_ receptor knockout mice control mice [33]. The overexpression of the CB_2_ receptor in the central nervous system also disturbs glucose homeostasis. Reduced glucose tolerance and prolonged fasting hyperglycemia were observed in 18- and 24-week-old animals with CB2 receptor overexpression in the brain [32]. However, after a single ip administration of JWH133, the same group demonstrated decreased glucose intolerance in wild type animals. Our findings are consistent with studies of global CB_2_ receptor gene deletion and the peripheral administration of drugs that modulate CB_2_ signaling that show that CB_2_ receptor activation promotes an imbalance in glucose homeostasis, leading to increased glycemia.

As expected, we observed that a HFD increased microgliosis in the ARC [16] and PVN. Similarly, increased microgliosis in the PVN and subfornical organ (SFO) was also reported in animals fed a HFD for 8 weeks [39]. These areas are important to the control of the neuroendocrine axis and autonomic nervous system and can contribute to the cardiovascular and glycemic changes observed in obesity. Nevertheless, the reduced microgliosis in the ARC of HU308-treated animals had no effect on the obesity induced by HFD intake. This result is interesting since some studies suggest that inflammation of the hypothalamus precedes the onset of obesity [40]. Several studies have indicated that hypothalamic inflammation favors the accumulation of fat associated with resistance to leptin actions [16,41,42]. Thus, it would be reasonable to suppose that with the reduced microgliosis in the ARC, leptin signaling would be restored and that the increased leptin tone would favor weight loss. However, leptin signaling in the PVN, ARC and VMH was not changed, as observed by the preserved STAT3 phosphorylation after CB_2_ modulation.

In mice with HFD-induced obesity, a reduction in hypothalamic inflammation was associated with improved glycemia [14,43]. Specifically, the reduced ARC inflammation by ARC-restricted TLR4 knockdown protects HFD-fed animals from impaired glucose homeostasis and peripheral insulin resistance [44]. Remarkably, we observed a worsening in glucose tolerance in animals with reduced microgliosis in the ARC that was induced by the icv infusion of HU308. An increase in basal glycemia as well as during GTT was also observed in mice with increased CB_2_ expression in the ARC and VMH [32]. Interestingly, we observed increased microgliosis in the VMH of lean and obese mice treated with HU308. The VMH is essential to the control of the counterregulatory response to hypoglycemia [45]. Recently, it was reported that mice with genetic disruption of glutamate release from steroidogenic factor neurons (SF1) in the VMH have attenuated recovery from hypoglycemia induced by insulin treatment [46] and, chemogenetic activation of this neuronal subpopulation induces hyperglycemia and enhances the counterregulatory response to glucopenia [47]. Taking this information into account, the activation of VMH could be involved in the increase in hyperglycemia that was observed in the HFD-HU308 group. Finally, the preserved control of glucose homeostasis (e.g., insulin action and secretion) could be underly the appropriated GTT response observed in control diet group treated with HU308.

Some studies have observed increased reactive astrocyte gliosis (activation and recruitment) in response to high-fat diet consumption [16,48]. The increase in the number of astrocytes due to the consumption of a HFD seems to be dependent on the duration of diet exposure. Thus, consumption of a HFD for up to 2 weeks increases recruitment and promotes changes in the morphology of ARC astrocytes [16]. Buckman and colleagues (2013) observed hypertrophy and hyperplasia of astrocytes in the hypothalamus after 20 weeks of HFD consumption [48]. However, Thaler et al. 2012 observed a transitory quiescence of the astrocytic process of gliosis after 3 weeks of HFD consumption and the restart of this process again after the 8th month of HFD consumption. Considering the neuroprotective role played by astrocytes during the inflammatory responses [49,50], brain damage (e.g., evidenced by neuronal death) and deficits in synaptic plasticity caused by HFD [16,51], we expected to observe astrogliosis in the hypothalamus. Therefore, absence of changes in astrogliosis may be due to the temporal window of the quiescent activity of astrocytes during prolonged HFD consumption.

Interestingly, we also observed that the CB_2_ receptor agonism with HU308 increases astrogliosis in the VMH only in obese animals. The effects of HU308 on astrocyte activity are similar to other results that have been reported in the literature [52,53]. As previously described, VMH is critical for the control of glucose homeostasis. Interestingly, astrocytes are important to the maintenance of the extracellular homeostasis and are involved in the regulation of synaptic transmission [54] and consequently in the modulation of neuronal activity. Indeed, activation of astrocytes induces long-term potentiation and memory acquisition [55]. Furthermore, the effects of astrocytes on neuronal activity are associated, at least in part, with synthesis and release of vasoactive substances [56], which can change local microvascular diameters and, as a result, modulate blood flow [57]. However, additional studies aimed to investigate the synthesis/secretion of inflammatory/vasoactive substances must be performed to clarify the role of CB_2_ receptor signaling in the control of astrocytic activity in the VMH, particularly in obese animals.

CB_2_ receptor signaling, leptin and inflammatory stimuli can activate the ERK pathway [20,30,58]. Although hypothalamic ERK signaling has an important effect on energy and glucose homeostasis [31,59], no evident effect of ERK phosphorylation was observed in the different hypothalamic nuclei after the modulation of CB_2_ receptor signaling. It is important to highlight that the immunostaining for pERK performed by us clearly indicated the recruitment of this pathway in neurons and not in immune cells, as there was clear staining in the bodies of neurons and not punctual glial staining. Some previous studies have indicated that there is CB_2_ receptor expression on neurons and other studies have indicated that it is expressed on glial cells [35,60,61,62]. However, the data of ERK phosphorylation described in our study suggest that the recruitment of this pathway in the PVN, ARC and VMH is not involved in the responses observed by us.

## 5. Conclusions

Given the close association between hypothalamic inflammation and obesity, as well as the anti-inflammatory CB_2_ receptor properties, as evidenced by the reduced microgliosis in the ARC, we cannot rule out the possibility that continuous infusion of CB_2_ receptor ligands for a longer period of time, longer than the one used in our study, could improve obesity caused by HFD intake. Moreover, our data indicate that central CB_2_ signaling modulates glucose homeostasis and glial reactivity in obesogenic conditions, irrespective of changes in body weight.

## Data Availability

Not applicable.

## References

[1] Fontaine K.R., Redden D.T., Wang C., Westfall A.O., Allison D.B. (2003). Years of life lost due to obesity. JAMA.

[2] Blüher M. (2019). Obesity: Global epidemiology and pathogenesis. Nat. Rev. Endocrinol..

[3] Camacho S., Ruppel A. (2017). Is the calorie concept a real solution to the obesity epidemic?. Glob. Health Action.

[4] Elmquist J.K., Coppari R., Balthasar N., Ichinose M., Lowell B.B. (2005). Identifying hypothalamic pathways controlling food intake, body weight, and glucose homeostasis. J. Comp. Neurol..

[5] Roh E., Song D.K., Kim M.S. (2016). Emerging role of the brain in the homeostatic regulation of energy and glucose metabolism. Exp. Mol. Med..

[6] Hetherington A.W., Ranson S.W. (1942). The relation of various hypothalamic lesions to adiposity in the rat. J. Comp. Neurol..

[7] Khodai T., Luckman S.M. (2021). Ventromedial Nucleus of the Hypothalamus Neurons Under the Magnifying Glass. Endocrinology.

[8] Campbell J.N., Macosko E.Z., Fenselau H., Pers T.H., Lyubetskaya A., Tenen D., Goldman M., Verstegen A.M., Resch J.M., McCarroll S.A. (2017). A molecular census of arcuate hypothalamus and median eminence cell types. Nat. Neurosci..

[9] Lowell B.B. (2019). New Neuroscience of Homeostasis and Drives for Food, Water, and Salt. N. Engl. J. Med..

[10] Li M.M., Madara J.C., Steger J.S., Krashes M.J., Balthasar N., Campbell J.N., Resch J.M., Conley N.J., Garfield A.S., Lowell B.B. (2019). The Paraventricular Hypothalamus Regulates Satiety and Prevents Obesity via Two Genetically Distinct Circuits. Neuron.

[11] Jiang Z., Rajamanickam S., Justice N.J. (2019). CRF signaling between neurons in the paraventricular nucleus of the hypothalamus (PVN) coordinates stress responses. Neurobiol. Stress.

[12] Smith S.M., Vale W.W. (2006). The role of the hypothalamic-pituitary-adrenal axis in neuroendocrine responses to stress. Dialogues Clin. Neurosci..

[13] Tsigos C., Chrousos G.P. (2002). Hypothalamic-pituitary-adrenal axis, neuroendocrine factors and stress. J. Psychosom. Res..

[14] Jais A., Brüning J.C. (2017). Hypothalamic inflammation in obesity and metabolic disease. J. Clin. Investig..

[15] De Souza C.T., Araujo E.P., Bordin S., Ashimine R., Zollner R.L., Boschero A.C., Saad M.J., Velloso L.A. (2005). Consumption of a fat-rich diet activates a proinflammatory response and induces insulin resistance in the hypothalamus. Endocrinology.

[16] Thaler J.P., Yi C.X., Schur E.A., Guyenet S.J., Hwang B.H., Dietrich M.O., Zhao X., Sarruf D.A., Izgur V., Maravilla K.R. (2012). Obesity is associated with hypothalamic injury in rodents and humans. J. Clin. Investig..

[17] Turnbull A.V., Rivier C.L. (1999). Regulation of the hypothalamic-pituitary-adrenal axis by cytokines: Actions and mechanisms of action. Physiol. Rev..

[18] Di Marzo V., Ligresti A., Cristino L. (2009). The endocannabinoid system as a link between homoeostatic and hedonic pathways involved in energy balance regulation. Int. J. Obes..

[19] Cassano T., Calcagnini S., Pace L., De Marco F., Romano A., Gaetani S. (2017). Cannabinoid Receptor 2 Signaling in Neurodegenerative Disorders: From Pathogenesis to a Promising Therapeutic Target. Front. Neurosci..

[20] Howlett A.C., Barth F., Bonner T.I., Cabral G., Casellas P., Devane W.A., Felder C.C., Herkenham M., Mackie K., Martin B.R. (2002). International Union of Pharmacology. XXVII. Classification of cannabinoid receptors. Pharmacol. Rev..

[21] Chang Y.H., Lee S.T., Lin W.W. (2001). Effects of cannabinoids on LPS-stimulated inflammatory mediator release from macrophages: Involvement of eicosanoids. J. Cell. Biochem..

[22] Facchinetti F., Del Giudice E., Furegato S., Passarotto M., Leon A. (2003). Cannabinoids ablate release of TNFalpha in rat microglial cells stimulated with lypopolysaccharide. Glia.

[23] Zoppi S., Madrigal J.L., Caso J.R., García-Gutiérrez M.S., Manzanares J., Leza J.C., García-Bueno B. (2014). Regulatory role of the cannabinoid CB2 receptor in stress-induced neuroinflammation in mice. Br. J. Pharmacol..

[24] Verty A.N., Stefanidis A., McAinch A.J., Hryciw D.H., Oldfield B. (2015). Anti-Obesity Effect of the CB2 Receptor Agonist JWH-015 in Diet-Induced Obese Mice. PLoS ONE.

[25] Bermudez-Silva F.J., Sanchez-Vera I., Suárez J., Serrano A., Fuentes E., Juan-Pico P., Nadal A., Rodríguez de Fonseca F. (2007). Role of cannabinoid CB2 receptors in glucose homeostasis in rats. Eur. J. Pharmacol..

[26] Deveaux V., Cadoudal T., Ichigotani Y., Teixeira-Clerc F., Louvet A., Manin S., Nhieu J.T., Belot M.P., Zimmer A., Even P. (2009). Cannabinoid CB2 receptor potentiates obesity-associated inflammation, insulin resistance and hepatic steatosis. PLoS ONE.

[27] Ghanbari M.M., Joneidi M., Kiani B., Babaie J., Sayyah M. (2020). Cannabinoid receptors and the proconvulsant effect of toxoplasmosis in mice. Microb. Pathog..

[28] Franklin K., Paxinos G. (2008). The Mouse Brain in Stereotaxic Coordinates, Compact.

[29] Borges Bde C., Rorato R., Uchoa E.T., Marangon P., da Silva G.S., de Paula F.J., Branco L.G., Antunes-Rodrigues J., Elias L.L. (2014). High-fat diet induces site-specific unresponsiveness to LPS-stimulated STAT3 activation in the hypothalamus. Am. J. Physiol. Regul. Integr. Comp. Physiol..

[30] Dhopeshwarkar A., Mackie K. (2014). CB2 Cannabinoid receptors as a therapeutic target-what does the future hold?. Mol. Pharmacol..

[31] Rahmouni K., Sigmund C.D., Haynes W.G., Mark A.L. (2009). Hypothalamic ERK mediates the anorectic and thermogenic sympathetic effects of leptin. Diabetes.

[32] Romero-Zerbo S.Y., Garcia-Gutierrez M.S., Suárez J., Rivera P., Ruz-Maldonado I., Vida M., Rodriguez de Fonseca F., Manzanares J., Bermúdez-Silva F.J. (2012). Overexpression of cannabinoid CB2 receptor in the brain induces hyperglycaemia and a lean phenotype in adult mice. J. Neuroendocrinol..

[33] Agudo J., Martin M., Roca C., Molas M., Bura A.S., Zimmer A., Bosch F., Maldonado R. (2010). Deficiency of CB2 cannabinoid receptor in mice improves insulin sensitivity but increases food intake and obesity with age. Diabetologia.

[34] Schmitz K., Mangels N., Häussler A., Ferreirós N., Fleming I., Tegeder I. (2016). Pro-inflammatory obesity in aged cannabinoid-2 receptor-deficient mice. Int. J. Obes..

[35] Onaivi E.S., Carpio O., Ishiguro H., Schanz N., Uhl G.R., Benno R. (2008). Behavioral effects of CB2 cannabinoid receptor activation and its influence on food and alcohol consumption. Ann. N. Y. Acad. Sci..

[36] Tilg H., Moschen A.R. (2006). Adipocytokines: Mediators linking adipose tissue, inflammation and immunity. Nat. Rev. Immunol..

[37] Rom S., Persidsky Y. (2013). Cannabinoid receptor 2: Potential role in immunomodulation and neuroinflammation. J. Neuroimmune Pharmacol..

[38] Yang P., Wang L., Xie X.Q. (2012). Latest advances in novel cannabinoid CB(2) ligands for drug abuse and their therapeutic potential. Future Med. Chem..

[39] De Kloet A.D., Pioquinto D.J., Nguyen D., Wang L., Smith J.A., Hiller H., Sumners C. (2014). Obesity induces neuroinflammation mediated by altered expression of the renin-angiotensin system in mouse forebrain nuclei. Physiol. Behav..

[40] Dorfman M.D., Thaler J.P. (2015). Hypothalamic inflammation and gliosis in obesity. Curr. Opin. Endocrinol. Diabetes Obes..

[41] Kleinridders A., Schenten D., Könner A.C., Belgardt B.F., Mauer J., Okamura T., Wunderlich F.T., Medzhitov R., Brüning J.C. (2009). MyD88 signaling in the CNS is required for development of fatty acid-induced leptin resistance and diet-induced obesity. Cell Metab..

[42] Milanski M., Degasperi G., Coope A., Morari J., Denis R., Cintra D.E., Tsukumo D.M., Anhe G., Amaral M.E., Takahashi H.K. (2009). Saturated fatty acids produce an inflammatory response predominantly through the activation of TLR4 signaling in hypothalamus: Implications for the pathogenesis of obesity. J. Neurosci..

[43] Ullah R., Rauf N., Nabi G., Yi S., Yu-Dong Z., Fu J. (2021). Mechanistic insight into high-fat diet-induced metabolic inflammation in the arcuate nucleus of the hypothalamus. Biomed. Pharmacother..

[44] Zhao Y., Li G., Li Y., Wang Y., Liu Z. (2017). Knockdown of Tlr4 in the Arcuate Nucleus Improves Obesity Related Metabolic Disorders. Sci. Rep..

[45] Shimazu T., Fukuda A., Ban T. (1966). Reciprocal influences of the ventromedial and lateral hypothalamic nuclei on blood glucose level and liver glycogen content. Nature.

[46] Tong Q., Ye C., McCrimmon R.J., Dhillon H., Choi B., Kramer M.D., Yu J., Yang Z., Christiansen L.M., Lee C.E. (2007). Synaptic glutamate release by ventromedial hypothalamic neurons is part of the neurocircuitry that prevents hypoglycemia. Cell Metab..

[47] Meek T.H., Nelson J.T., Matsen M.E., Dorfman M.D., Guyenet S.J., Damian V., Allison M.B., Scarlett J.M., Nguyen H.T., Thaler J.P. (2016). Functional identification of a neurocircuit regulating blood glucose. Proc. Natl. Acad. Sci. USA.

[48] Buckman L.B., Thompson M.M., Moreno H.N., Ellacott K.L. (2013). Regional astrogliosis in the mouse hypothalamus in response to obesity. J. Comp. Neurol..

[49] Pekny M., Nilsson M. (2005). Astrocyte activation and reactive gliosis. Glia.

[50] Sofroniew M.V. (2009). Molecular dissection of reactive astrogliosis and glial scar formation. Trends Neurosci..

[51] Horvath T.L., Sarman B., García-Cáceres C., Enriori P.J., Sotonyi P., Shanabrough M., Borok E., Argente J., Chowen J.A., Perez-Tilve D. (2010). Synaptic input organization of the melanocortin system predicts diet-induced hypothalamic reactive gliosis and obesity. Proc. Natl. Acad. Sci. USA.

[52] Köfalvi A., Lemos C., Martín-Moreno A.M., Pinheiro B.S., García-García L., Pozo M.A., Valério-Fernandes Â., Beleza R.O., Agostinho P., Rodrigues R.J. (2016). Stimulation of brain glucose uptake by cannabinoid CB2 receptors and its therapeutic potential in Alzheimer’s disease. Neuropharmacology.

[53] Sheng W.S., Hu S., Min X., Cabral G.A., Lokensgard J.R., Peterson P.K. (2005). Synthetic cannabinoid WIN55, 212–2 inhibits generation of inflammatory mediators by IL-1beta-stimulated human astrocytes. Glia.

[54] Perea G., Sur M., Araque A. (2014). Neuron-glia networks: Integral gear of brain function. Front. Cell. Neurosci..

[55] Adamsky A., Kol A., Kreisel T., Doron A., Ozeri-Engelhard N., Melcer T., Refaeli R., Horn H., Regev L., Groysman M. (2018). Astrocytic Activation Generates De Novo Neuronal Potentiation and Memory Enhancement. Cell.

[56] Volterra A., Meldolesi J. (2005). Astrocytes, from brain glue to communication elements: The revolution continues. Nat. Rev. Neurosci..

[57] Attwell D., Buchan A.M., Charpak S., Lauritzen M., Macvicar B.A., Newman E.A. (2010). Glial and neuronal control of brain blood flow. Nature.

[58] Coppari R., Bjørbæk C. (2012). Leptin revisited: Its mechanism of action and potential for treating diabetes. Nat. Rev. Drug Discov..

[59] Toda C., Shiuchi T., Kageyama H., Okamoto S., Coutinho E.A., Sato T., Okamatsu-Ogura Y., Yokota S., Takagi K., Tang L. (2013). Extracellular signal-regulated kinase in the ventromedial hypothalamus mediates leptin-induced glucose uptake in red-type skeletal muscle. Diabetes.

[60] Núñez E., Benito C., Pazos M.R., Barbachano A., Fajardo O., González S., Tolón R.M., Romero J. (2004). Cannabinoid CB2 receptors are expressed by perivascular microglial cells in the human brain: An immunohistochemical study. Synapse.

[61] Romero-Sandoval E.A., Horvath R., Landry R.P., DeLeo J.A. (2009). Cannabinoid receptor type 2 activation induces a microglial anti-inflammatory phenotype and reduces migration via MKP induction and ERK dephosphorylation. Mol. Pain.

[62] Van Sickle M.D., Duncan M., Kingsley P.J., Mouihate A., Urbani P., Mackie K., Stella N., Makriyannis A., Piomelli D., Davison J.S. (2005). Identification and functional characterization of brainstem cannabinoid CB2 receptors. Science.

